# Supplementary material for: Evolved differences in larval social behavior mediated by novel pheromones
Source: eLife. 2014 Dec 12;3:e04205. doi: 10.7554/eLife.04205 (PMC4270068; doi:10.7554/eLife.04205)
Supplement: Supplementary file 1. — DOI: http://dx.doi.org/10.7554/eLife.04205.017 [file elife04205s002.docx]

% Tracker and Prefence Index calculation

% initially based on tastPro_v1.m code

% by Alex Gomez-Marin (Louis lab, EMBL-CRG, Barcelona)

% 11th June 2010

%

% MATLAB language. Toolboxes needed: Image processing toolbox.

clear

close all

dataDir0='';

%careful with name here

file=''

movArray=dir(strcat(dataDir0,file));

maxMov=length(movArray);

for movNum=1:maxMov

clear filesArray

clear totalFrames

clear maxFile

clear sequence

clear XYpositions

clear i0

CurrentMovie=movArray(movNum).name

dataDir=strcat(dataDir0,'/', CurrentMovie);

cd(dataDir);

% read image background (very important that it is really static during the experiment)

i0=imread('background.bmp');

% manually determine the four corners of the arena

%

% ARENA1: click top left and bottom right

figure,

imshow(i0)

text(50,50,'CLICK ON THE LEFT-TOP AND RIGHT-BOTTOM CORNER OF THE LEFT ARENA')

axis equal

set(gca,'xtick',[],'ytick',[])

[xTL,yTL]=ginput(1);

%[xTR,yTR]=ginput(1);

[xBR,yBR]=ginput(1);

%[xBL,yBL]=ginput(1);

xTR=xBR;yTR=yTL;xBL=xTL;yBL=yBR;

% we write down the position (in pixels! still we need conversion to mm)

% plotting the background landmarks to make sure they make sense

close all

figure,

imshow(i0)

text(50,50,'IF OK, PRESS ANY KEY')

hold on

plot([xTL xBL],[yTL yBL],'-k')

plot([xTR xBR],[yTR yBR],'-k')

plot([xTL xTR],[yTL yTR],'-k')

plot([xBL xBR],[yBL yBR],'-k')

wellspaceX=(xTR-xTL)*(1/4.3); %4x1"square wells + 3x0.1" borders

wellspaceY=(yBL-yTL)*(1/4.3);

%mask over outside of arena

mask=roipoly(i0, [xTL xTR xBR xBL],[yTL yTR yBR yBL]); %c columns, r rows

mask2=logical(abs(1-mask));

for i=1:4

xchL(i)=xTL+((i-1)*wellspaceX)+((i-1)*(wellspaceX*0.1));

% LEFT SIDE: xTL +(i-1)wells + (i-1)spacers

xchR(i)=xTL+(i*wellspaceX)+((i-1)*(wellspaceX*0.1));

% RIGHT SIDE: xTL + (i)wells + (i-1)spacers

ychT(i)=yTL+((i-1)*wellspaceY)+((i-1)*(wellspaceY*0.1));

% TOP SIDE: xTL +(i-1)wells + (i-1)spacers

ychB(i)=yTL+(i*wellspaceY)+((i-1)*(wellspaceY*0.1));

% BOTTOM SIDE: xTL + (i)wells + (i-1)spacers

end

%draw to check

for i=1:4,

rectangle('Position',[xchL(1,i), ychT(1,1), wellspaceX, wellspaceY], 'EdgeColor','r');

rectangle('Position',[xchL(1,i), ychT(1,2), wellspaceX, wellspaceY], 'EdgeColor','r');

rectangle('Position',[xchL(1,i), ychT(1,3), wellspaceX, wellspaceY], 'EdgeColor','r');

rectangle('Position',[xchL(1,i), ychT(1,4), wellspaceX, wellspaceY], 'EdgeColor','r');

end

%END OF INSERTION

% prepare the array of frame tags to read them afterwards:

filesArray=dir(strcat(dataDir,'/1*.bmp'));

maxFile=length(filesArray);

% total frames available

totalFrames=maxFile;

%modified for 5.5 minute movie

%frame sequence we wish to analyze

%change back to 300

sequence=1:1:300;

% example of only 1000 frames, every 5 of them:

%sequence=1:5:1000;

tic

countFile=0;

for file=sequence

countFile=countFile+1;

%try

filename=filesArray(file).name;

i1=imread(filename);

% this part is crucial for the image processing to work:

% 1. substract background

ix=i0-i1;

%threshold loop added 020711

threshold=0.14;

numobj=0;

while numobj==0;

% 2. theshold image (different illuminations may need tuning of threshold)

threshold=threshold-(0.005);

iw=im2bw(ix,threshold);

%APPLY ARENA MASK (050211)

im=iw-mask2;

im=(im+abs(im))/2; %get rid of negative values

% need to blur to avoid larva splitted in two

%this may add more noise to this analysis.. test this without

%dilation

imdil=imdilate(im,ones(2));

%imdil=bwmorph(im,'dilate',2); old, broken after 2013a installation

%(08/19/13)

% detect objects?

[L,numobj] =bwlabel(imdil,8);

threshold

end

for i=1:4

%total larval area

total_area(countFile,movNum)=sum(sum(imdil));

%larval area in wells

larval_dist(i*4-3,countFile,movNum)=sum(sum(imdil(ychT(1,1):ychB(1,1),xchL(1,i):xchR(1,i))))/total_area(countFile,movNum);

larval_dist(i*4-2,countFile,movNum)=sum(sum(imdil(ychT(1,2):ychB(1,2),xchL(1,i):xchR(1,i))))/total_area(countFile,movNum);

larval_dist(i*4-1,countFile,movNum)=sum(sum(imdil(ychT(1,3):ychB(1,3),xchL(1,i):xchR(1,i))))/total_area(countFile,movNum);

larval_dist(i*4,countFile,movNum)=sum(sum(imdil(ychT(1,4):ychB(1,4),xchL(1,i):xchR(1,i))))/total_area(countFile,movNum);

end

%sum similar treatments

%CHANGE THIS TO FIT ASSAY CONDITIONS

%this is for a staggered checkerboard

%fit movie label into structure!!

data.condition1(countFile,movNum)=sum(larval_dist([2 4 5 7 10 12 13 15],countFile,movNum));

data.condition2(countFile,movNum)=sum(larval_dist([1 3 6 8 9 11 14 16],countFile,movNum));

data.runPI(countFile,movNum)=((data.condition2(countFile,movNum)-data.condition1(countFile,movNum))/(data.condition2(countFile,movNum)+data.condition1(countFile,movNum)));

end

end

%I want this to average each condition (i) across all movies for all times

%(t)

for t=1:300

data.mean_condition1(t)=mean(data.condition1(t,:));

data.mean_condition2(t)=mean(data.condition2(t,:));

data.PI_condition1(t)=(data.mean_condition1(t)-data.mean_condition1(t))/(data.mean_condition1(t)+data.mean_condition1(t));

data.PI_condition2(t)=(data.mean_condition2(t)-data.mean_condition1(t))/(data.mean_condition2(t)+data.mean_condition1(t));

data.condition2_p(t)=ranksum(data.condition1(t,:),data.condition2(t,:));

end

%integration

for i=1:maxMov

for t=1:300

data.movPI_condition1(t,i)=(data.condition1(t,i)-data.condition1(t,i))/(data.condition1(t,i)+data.condition1(t,i));

%should be 0; just checking

data.movPI_condition2(t,i)=(data.condition2(t,i)-data.condition1(t,i))/(data.condition2(t,i)+data.condition1(t,i));

end

%integrating the area under the entire PI curve for each movie

data.int_PI1(i)=trapz(data.movPI_condition1(:,i));

data.int_PI2(i)=trapz(data.movPI_condition2(:,i));

%integrating the area after the first 30 seconds

data.int30_PI1(i)=trapz(data.movPI_condition1(30:300,i));

data.int30_PI2(i)=trapz(data.movPI_condition2(30:300,i));

end

figure(6)

subplot(2,1,1)

plot(data.mean_condition1,'Color', 'r' );hold on %control

plot(data.mean_condition2,'Color', 'b');hold on %high

subplot(2,1,2)

plot(data.PI_condition1,'Color', 'r' );hold on %control

plot(data.PI_condition2,'Color', 'b');hold on %high

saveas(gcf, 'summary', 'fig')

for i=1:maxMov

figure(2);hold on

subplot(maxMov,1,i);

plot(data.condition1(:,i),'Color', 'r' );hold on %control

plot(data.condition2(:,i),'Color', 'b');hold on %high

end

saveas(gcf, 'individuals', 'fig')

savefile=strcat(CurrentMovie, date,'.mat');

save(savefile,'data')
